# Favorable response to second-line atezolizumab and bevacizumab following progression on nivolumab in advanced hepatocellular carcinoma: A case report demonstrating that anti-VEGF therapy overcomes resistance to checkpoint inhibition

**DOI:** 10.1097/MD.0000000000026471

**Published:** 2021-06-25

**Authors:** Brandon Swed, Kara Ryan, Omar Gandarilla, Manish A. Shah, Gagandeep Brar

**Affiliations:** a Division of Hematology and Medical Oncology; b Internal Medicine Residency Program, Department of Medicine; c Center for Advanced Digestive Care, Weill Cornell Medicine/New York-Presbyterian Hospital, New York, NY.

**Keywords:** anti-VEGF therapy, case report, hepatocellular carcinoma, immune checkpoint inhibition, immunotherapy resistance

## Abstract

**Rationale:**

Advanced hepatocellular carcinoma (HCC) remains a deadly disease in part due to decades of limited therapeutic options. With recent advances in our understanding of the tumor biology, several promising treatment strategies involving targeted and immunotherapies have emerged. However, enhancing their modest efficacy in HCC and other gastrointestinal malignancies is essential to improving survival.

**Patient concerns:**

A man in his late 50s with a history of type 2 diabetes mellitus and morbid obesity initially presented with progressive abdominal pain and anorexia prompting an abdominal computed tomography scan that revealed a large solitary liver mass with extensive local involvement.

**Diagnoses:**

Although there were features consistent with a primary gastric tumor on subsequent endoscopic evaluation leading to early diagnostic uncertainty, his clinical picture, including a dominant liver mass, immunohistochemical staining profile, and significantly elevated alpha fetoprotein ultimately favored HCC.

**Interventions:**

The patient received palliative systemic therapy with infusional fluorouracil for a presumed gastric primary, however restaging scans after 3 cycles demonstrated disease progression. The consensus from a multidisciplinary discussion was that his pathology was more consistent with primary HCC. He was subsequently started on nivolumab with a partial response, although after 5 months, he progressed prompting initiation of second-line atezolizumab and bevacizumab with a favorable response.

**Outcomes:**

The addition of atezolizumab and bevacizumab led to a sustained biochemical and radiographic response that appeared to overcome the resistance to nivolumab monotherapy. Aside from several mild immune-related adverse effects, his quality of life has greatly improved and he has tolerated treatment well to date.

**Lessons:**

Our findings suggest that vascular endothelial growth factor inhibition can overcome resistance to checkpoint inhibition in advanced HCC by resulting in a unique synergy that has never before been described in patients. The biological rationale for this response is likely attributable to the immunomodulatory effects of antiangiogenic agents, promoting an immunostimulatory microenvironment that can be exploited by immune checkpoint inhibitors for more effective antitumor activity. Given the considerable benefit patients may derive following progression on first-line treatment, it is important to consider this strategic combination of therapies which can ultimately lead to improved patient outcomes.

## 1 Introduction

Hepatocellular carcinoma (HCC), the most common form of primary liver cancer, is a major contributor to the worldwide cancer burden. With a 5-year survival rate of 18% across all stages, it remains the third leading cause of cancer-related death globally.^[1]^ Although incidence of HCC has increased over the past several decades, until recently, therapeutic advances have largely remained stagnant and clinical outcomes remain poor.

Although surgery, including resection and liver transplantation, and ablative techniques are curable in select cases with early-stage disease, recurrence rates remain high. Alternative treatment options include locoregional therapy in the form of embolization and radiation. In unresectable or advanced tumors with extrahepatic spread, standard of care involves systemic therapy.^[2]^ For decades, sorafenib, an oral multi-tyrosine kinase inhibitor (TKI), was the only FDA-approved treatment for patients with advanced HCC based on a modest survival benefit when compared with placebo.^[3]^ Lenvatinib, a similar oral multi-TKI, was recently approved as an alternative first-line therapy based on noninferiority when compared with sorafenib.^[4]^ In addition, several other multi-target inhibitors, including regorafenib, cabozantinib, and ramucirumab, are approved in the second-line setting.^[5–7]^

**Figure 1:**
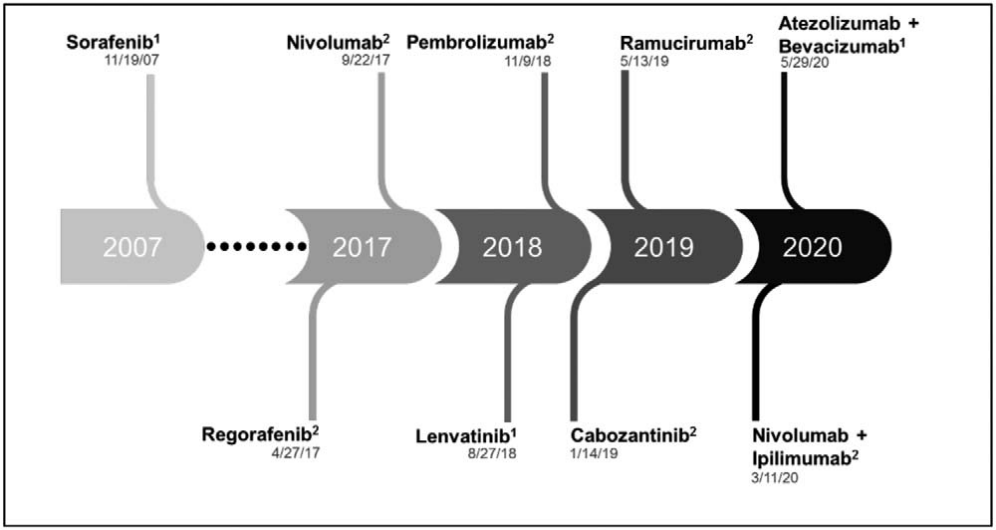
Schematic timeline of recent United States Food and Drug Administration (FDA) approvals for systemic therapy in advanced HCC, with dates of approval listed below each drug name. ^1^Approved in the first-line setting. ^2^Approved for subsequent-line therapy.

More recently, there has been a significant shift in the treatment landscape of HCC, as we better understand the biology of these tumors. In addition to molecularly targeted agents, immune checkpoint inhibitors have demonstrated favorable outcomes in patients with HCC and are approved in the advanced stage setting. For example, nivolumab, a PD-1 inhibitor, was shown to have a survival benefit as second-line treatment. However, when nivolumab was evaluated in the first-line setting, although it had a favorable toxicity profile, there was no significant overall survival benefit when compared with sorafenib.^[8]^ Similarly, the PD-1 inhibitor pembrolizumab was shown to be safe and effective in previously treated patients with advanced HCC, although, as observed with nivolumab, survival benefit did not reach statistical significance.^[9]^ In addition, the combination of nivolumab and ipilimumab, an anti-CTLA-4 antibody, was recently granted accelerated approval in the second-line setting based on promising overall survival data.^[10]^ A timeline depicting the most recent systemic therapy approvals for advanced HCC is shown in Figure 1.

A breakthrough in the use of immunotherapy in the first-line setting came with the combination of atezolizumab, a PD-L1 inhibitor, and bevacizumab, a VEGF inhibitor. This combination was recently demonstrated to have a superior response rate, progression-free survival, and overall survival benefit when compared with sorafenib in treatment-naive patients with advanced HCC.^[11]^ A biological rationale for these findings can be drawn from evolving data suggesting that selective and multikinase inhibitors have an immunomodulatory effect on the tumor microenvironment.^[12]^ Specifically, antiangiogenic agents, including VEGF inhibitors, have been shown to counter local immunosuppressive effects by enhancing antigen presentation and immune effector cells and by downregulating the activity of several immunosuppressive mediators, including regulatory T cells, myeloid-derived suppressor cells, and tumor-associated macrophages. Coadministration of immune checkpoint inhibitors can then harness this immunostimulatory microenvironment to more effectively target and destroy cancer cells.^[13]^ As VEGF and immune checkpoints govern distinct but complementary tumor immune responses, it is conceivable that dual blockade of these targets results in a synergistic effect that may overcome resistance to immune checkpoint inhibition alone. This unique synergy has never been demonstrated in patients before.

Here, we present a case of HCC with a favorable response to combination atezolizumab and bevacizumab following progression on single-agent nivolumab.

## 2 Case report

A man in his late 50s with a history of type 2 diabetes mellitus and morbid obesity initially presented in December 2019 with abdominal distension, poor appetite, and melena prompting an outpatient CT abdomen/pelvis that revealed a 12 cm solitary liver mass with extensive portal vein and inferior vena cava (IVC) tumor thrombus, and multiple enlarged abdominal lymph nodes. Although outpatient workup was underway, he presented to the emergency department with acute onset exertional dyspnea and was found to have multiple right-sided pulmonary emboli on chest CT. Follow-up transthoracic echocardiogram showed a large tumor with associated thrombus extending from the IVC into the right atrium, which was confirmed on cardiac MRI. Initial labs were notable for a mild transaminitis (AST 233 U/L, ALT 121 U/L, and ALP 339 U/L).

He subsequently underwent a CT-guided biopsy of the hepatic lesion and pathology showed a high-grade carcinoma. The tumor's immunohistochemical (IHC) staining profile was most consistent with a primary gastric or gastroesophageal junction tumor with hepatoid and neuroendocrine differentiation; however, HCC could not be excluded based on glypican-3 and HepPar1 positivity, although arginase staining was negative. Given the diagnostic uncertainty, he underwent an upper gastrointestinal (GI) endoscopy, which demonstrated a large fungating circumferential gastric fundus mass. Brushings of the mass showed glandular epithelial cells with cytologic and architectural atypia suspicious for a primary gastric adenocarcinoma. At this time, he was found to have a marked elevation of alpha fetoprotein (AFP) to 8716.5 ng/mL. Further molecular profiling of his tumor was notable for the following: HER-2 negative, mismatch repair (MMR) preserved, PD-L1 combined positive score (CPS) of 2% by IHC. Next-generation sequencing revealed no clinically actionable alterations.

While inpatient, he received 5 fractions of palliative radiation therapy directed at the portal vein and IVC tumor thrombi, abdominal lymphadenopathy, and gastric fundus mass. He was then initiated on palliative systemic therapy in early January 2020 with infusional fluorouracil (5-FU) and leucovorin. This was selected in lieu of the more myelosuppressive standard of care FOLFOX regimen given his severe thrombocytopenia with a nadir platelet count of 16 x 10^3^/μL, likely multifactorial in the setting of confirmed heparin-induced thrombocytopenia, underlying liver disease and splenomegaly. He received a total of 3 cycles; however, a restaging CT scan in mid-February revealed progression of disease in his lung and lymph nodes and stable solitary liver mass. His AFP continued to rise and peaked at 107,866 ng/mL.

Given biochemical and radiographic evidence of disease progression on gastric cancer directed therapy, the case was revisited at a multidisciplinary tumor board. The consensus was that his pathology was more consistent with stage 4 primary HCC and the treatment plan was modified accordingly. He proceeded with 5 fractions of stereotactic body radiation therapy to the hepatic lesion at the end of February 2020 followed by systemic therapy with nivolumab given its indication for HCC and his ineligibility for TKI therapy. After 3 months of treatment, imaging showed a partial response to therapy, with interval decrease in his hepatic, pulmonary and nodal disease.

**Figure 2:**
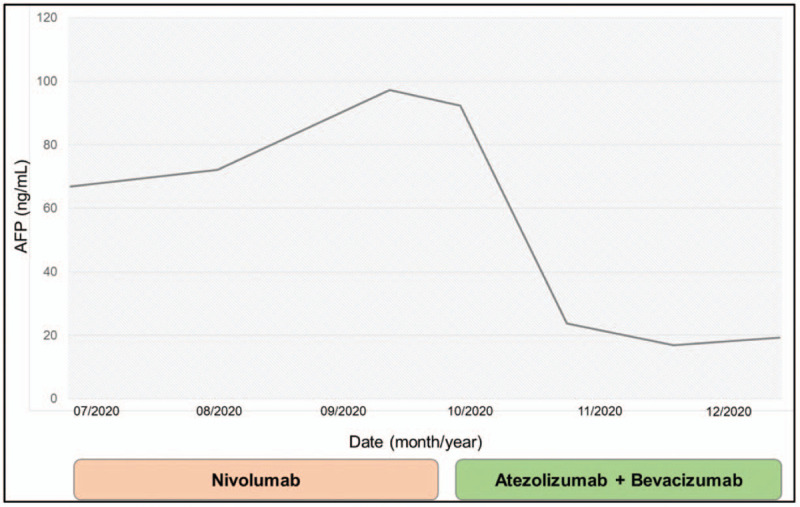
Serum alpha-fetoprotein (AFP) levels depicted in relation to course of systemic therapy.

He was maintained on nivolumab with a dramatic decrease in AFP to a nadir of 66.8 ng/mL by July 2020. His platelet count also recovered to 115 x 10^3^/μL during this time. However, over the next 2 months, his AFP began to slowly rise, and restaging scans demonstrated progressive nodal disease. In September 2020, he was initiated on combination therapy with atezolizumab and bevacizumab dosed every 3 weeks given its safety and efficacy profile and perceived benefit following progression on nivolumab monotherapy. AFP levels continued to downtrend (Fig. 2) and his scans from December 2020 showed a partial response with stable thoracic nodal disease burden and a decrease in size of his hepatic mass and abdominopelvic adenopathy. He experienced a sustained response for four months, after which time he was started on next-line lenvatinib for thoracic nodal disease progression, with a duration of follow-up of 8 months. Importantly, aside from several mild immune-related adverse effects including arthritis and lower back rash, his treatment was well-tolerated and his quality of life has greatly improved.

## 3 Discussion

Improving on the modest efficacy of checkpoint inhibition therapy in HCC and other GI malignancies is critical to improving patient survival. The combination of antiangiogenic agents and immune checkpoint inhibitors has recently gained traction as a practice-changing approach to the management of advanced HCC. Specifically, the combination of atezolizumab and bevacizumab was recently approved for use in patients with treatment-naive disease based on positive findings from the phase III IMbrave150 study.^[11]^ However, the optimal sequence strategy of first- and second-line systemic therapies still remains elusive. To our knowledge, the case presented here is the first report of atezolizumab and bevacizumab effectively used in the second-line setting following progression on single-agent anti-PD-1 therapy, and specifically suggests that VEGF inhibition can overcome resistance to checkpoint inhibition in HCC.

The biological rationale for this observed response is likely attributable to the immunomodulatory effects of this combination therapy on the tumor microenvironment. Growing evidence from preclinical data suggests that combining anti-angiogenic drugs and immune checkpoint inhibitors results in a reprogramming of the tumor microenvironment to become less immunosuppressive and more immunostimulatory through a variety of mechanisms.^[13]^ Specifically, targeted agents that inhibit VEGF activity result in increased antigen presentation by dendritic cells, activation of T cells, and downregulation of immunosuppressive cytokines and regulatory T cells. Furthermore, anti-VEGF antibodies normalize the tumor vasculature to promote effective infiltration of T cells into the tumor. Together with immune checkpoint inhibitors, there is a greater ability of T cells to attack tumor cells.^[12,13]^ As such, this combination therapy plausibly results in enhanced cancer immunity and antitumor effects. Our case report strongly suggests that anti-VEGF therapy was indeed able to overcome resistance to immune checkpoint inhibition. This therapeutic rationale has led to a number of ongoing clinical trials evaluating the efficacy of this combination therapy in advanced HCC (Table 1).

**Table 1:** Clinical trials combining immune checkpoint inhibitors with VEGF/tyrosine kinase inhibitors.

Trial	Current phase	Combination therapy	Molecular targets
IMbrave150^[11]^	Phase III	Atezolizumab + Bevacizumab	PD-L1 + VEGF
LEAP-002^[14]^	Phase III	Pembrolizumab + Lenvatinib	PD-1 + VEGFR1–3, FGFR1–4, PDGFRα, KIT, RET
RESCUE^[15]^	Phase II	Camrelizumab + Apatinib	PD-1 + VEGFR2
VEGF Liver 100^[16]^	Phase I	Avelumab + Axitinib	PD-L1 + VEGFR1–3
IMMUNIB^[17]^	Phase II	Nivolumab + Lenvatinib	PD-1 + VEGFR1–3, FGFR1–4, PDGFRα, KIT, RET
COSMIC-312^[18]^	Phase III	Atezolizumab + Cabozantinib	PD-L1 + VEGFR-1–3, KIT, MET, RET, among others

PD-1 = programmed cell death protein 1, PD-L = programmed cell death-ligand 1, VEGF = vascular endothelial growth factor. PDGFRα = platelet-derived growth factor receptor α, VEGFR1–3 = vascular endothelial growth factor receptors 1–3, FGFR1–4 = fibroblast growth factor receptors 1–4. KIT: proto-oncogene, receptor tyrosine kinase; RET: proto-oncogene, receptor tyrosine kinase; MET: proto-oncogene, receptor tyrosine kinase.

The advent of targeted and immunotherapy has ushered in a new era in the treatment of HCC. With better efficacy and side effect profiles, these agents have already begun to improve patient outcomes and quality of life. In particular, our findings suggest that patients may derive considerable benefit from atezolizumab and bevacizumab following progression on first-line treatment, especially in those who may not have had access to this combination upfront and may not be eligible for standard second-line therapies. Accordingly, thoughtful selection and strategic sequence of such combination therapies may further shift the therapeutic paradigm of this fatal malignancy. Further understanding of mechanisms of synergy and resistance are necessary, as are the identification of factors that may predict response to such therapies.

## Author contributions

**Conceptualization:** Brandon Swed, Manish A Shah, Gagandeep Brar.

**Data curation:** Brandon Swed, Kara Ryan, Omar Gandarilla.

**Formal analysis:** Manish A Shah.

**Investigation:** Brandon Swed, Kara Ryan, Omar Gandarilla, Gagandeep Brar.

**Supervision:** Manish A Shah, Gagandeep Brar.

**Visualization:** Brandon Swed, Manish A Shah.

**Writing – original draft:** Brandon Swed, Kara Ryan.

**Writing – review & editing:** Brandon Swed, Manish A Shah, Gagandeep Brar.
